# Congenital Hypothyroidism with Neurological and Respiratory Alterations: A Case Detected Using a Variable Diagnostic Threshold for TSH

**DOI:** 10.4274/jcrpe.448

**Published:** 2011-12-06

**Authors:** Jesús Barreiro, Jóse Ramón Alonso-Fernândez, Lidia Castro-Feijoo, Cristóbal Colón, Paloma Cabanas, Claudia Heredia, Luis Antonio Castaño, Carmen Gómez-Lado, M.Luz Couce, Manuel Pombo

**Affiliations:** 1 Hosp Clînico/Universidade de Santiago, Unidad de Endocrinologia Pediatrica, Crecimiento y Adolescencia Pediatrîa, Galicia, Spain; 2 Hosp Clînico/Universidade de Santiago de Compostela, Lab Metabolopatîas Pediatria, Galicia, Spain; 3 Hosp Clînico/Universidade de Santiago de Compostela, Neonatologîa, Pediatrîa, Galicia, Spain; 4 Hosp de Cruces Universidad del Paîs Vasco, Grupo de Investigación en Endocrinologîa y Diabetes, Vizczya, Vascongadas, Spain; 5 Hosp Clînico/Universidade de Santiago de Compostela, Unidad de Neuropediatria Pediatrîa, Galicia, Spain; +34 981950100+34 981950101joseramon.alonso@usc.esLab Metabolopatias. Hospital Clinico (CHUS). Choupana, s/n. E-15706 Santiago de Compostela, Spain

**Keywords:** congenital hypothyroidism, TSH cut-off, newborn screening, neurological pathology, respiratory pathology, TITF1/NKX2-1 gene

## Abstract

We report a case of congenital hypothyroidism (CH) with neurological and respiratory alterations due to a heterozygotic c.374-1G > A mutation of TITF1/NKX2-1. The hypothyroidism was detected using a neonatal screening protocol in which the thyroid stimulating hormone (TSH) threshold is re-set each day on the basis of within-day variability and between-day variation. In this case, the threshold on the day of the  initial analysis was 8.2 mIU/L, and the measured TSH level in heel-prick blood was 8.3 mIU/L.

**Conflict of interest:**None declared.

## INTRODUCTION

For application in the detection of congenital hypothyroidism (CH), we recently described an algorithm for calculating a diagnostic threshold for thyroid stimulating hormone (TSH) that is specific to each analytical run (1). In this previous report, we had  mentioned three cases of hypothyroidism that were detected using this variable threshold and which would not have been detected using the conventional threshold of 10 mIU/L. Here we describe one of these cases in greater detail.

## CASE REPORTS

In 2006, a neonatal screening blood spot taken from a newborn boy at age 3 days, and analyzed in our laboratory�8 days later, was found to have an Auto dissociation- enhanced lanthanide fluorescence immunoassay (DELFIA)-measured TSH concentration of 8.3 mIU/L in a run for which the�run-specific TSH threshold was 8.2 mlU/L.� Duplicate analyses of a second aliquots from the same spot yielded values of 8.2 and 9.4 mIU/L in a run with a threshold of 8.6 mIU/L. A second  paper-borne sample was requested, taken at age 18 days, and analyzed in duplicate upon arrival the following day. Whole blood TSH levels of 57.3 and 66.9 mIU/L indicated hypothyroidism, and were corroborated by analysis of a  conventional blood sample taken on the same day which showed a serum TSH of 126 mIU/L (reference range  0.35-5.50 mIU/L), a serum free thyroxine (fT4) of  0.75 ng/dL (reference range 0.08-1.87 ng/dL), a serum free triiodothyronine (fT3) of 3.55 pg/mL, and a serum thyroglobulin (Tg) of  529.0 ng/mL. 

 Thresholds are calculated in a spreadsheet with provision for input of ancillary information, such as calibration line data, that facilitates various analyses. 

At that stage (day 19), the child weighed 4410 g, measured 55.4 cm, and had a cranial circumference of 37.5 cm. The posterior fontanelle was lacking, the anterior measured 2x3 cm. Appearance of the face, muscle tone and chest auscultation were normal, no abdominal hernia was detected, and no jaundice was observed. Constipation during the first few days of life was reported to have been treated with an appropriate milk formula. Mother and child tested negative for antithyroid antibodies. A diagnosis of CH was made, and treatment with 14 μg/kg/day of levothyroxine (Eutirox 25 μg) was begun the same day.   

The patient was the first child of non-consanguineous parents. The father measured 1.84 m, the mother (age at menarche 17 years) 1.73 m. A brother of the mother had been treated for euthyroid goitre when adolescent, but there was no family history of mental retardation, deafness or autoimmune disease.  

Gestation had been supervised, incident-free and drug-free, except for diazepam which  had been used for insomnia. No iodated antiseptics had been used. Birth was by spontaneous vertex delivery at term, with no use of povidone-iodine or other iodine-bearing compounds. The infant  weighed 3. 590 g (62.5th percentile) at birth, measured 52.0 cm (82.5th percentile), and had an Apgar score of 9/10. During the first day, he received oxygen for transitory tachypnoea of the newborn. He was fed from birth with formula milk.

After 15 days of treatment, serum TSH concentration was 0.45 mIU/L and serum fT4 concentration 1.89 ng/dL. Progress was monitored in accordance with the protocol of the Spanish Society of Paediatric Endocrinology; levothyroxine dosage was successively adjusted to maintain serum TSH <3 mIU/L and serum fT4 >1.5 ng/dL. Between the initiation of treatment and age 3 years, the greatest known deviation from these targets was a serum TSH concentration of 8.64 mIU/L at age 6 months, accompanied by a serum fT4 concentration of 1.89 ng/dL.  

Up to age 3 years, the patient suffered numerous airway infections (bronchiolitis), and following an eventual diagnosis of bronchial hyperactivity, was treated with bronchodilators and corticoid inhalants. At age 18 months, his tonsils and adenoids were removed.

During this period, progressive motor retardation was detected. The patient was able to maintain an upright seated position alone at age 10 months, stood alone at age 12 months, and at age 16 months, walked with help, but with a very unstable gait and a broad walking base. At age 22 months, he walked unaided, but with a broad walking base, frequent alls, poor coordination; dystonia and mild general hypotonia were observed. Intelligence quotient was normal at age 12 months, and referential speech began at age 20 months.Evaluation of the motor retardation in the neuropaediatrics service showed normal cranial MRI, electromyography and karyotype findings.  In view of the general hypotonia of probable mixed central and peripheral origin and the absence of teleangiectasies or altered ocular motility, treatment with levodopa was begun, but was suspended upon the appearance of dyskinaesia.    

The diagnosis of hypothyroidism was reconfirmed at age 3 years: following the suspension of therapy, serum TSH rose to 50.9 mIU/L and fT4 fell to 0.95 ng/dL, while fT3 showed little alteration at 3.87 pg/mL. Ultrasonography showed a thyroid of normal homogeneity and echogenicity but small size, the right lobe measuring approximately 9x5x12 mm and the left lobe 5x3x10 mm. 99mTc gammagraphy confirmed cervical location and showed no significant alteration of the right lobe but virtually no contrast uptake in the left.

The combination of hypothyroidism, moderate respiratory problems and motor control problems suggested the possibility of TITF1/NKX2-1 haploinsufficiency (2). A requested genetic study revealed a heterozygotic c.374-1G >A mutation, resulting in loss of the exon 2 splicing site and truncation of the protein.

## DISCUSSION

Hypothyroidism is the commonest paediatric endocrine disorder and the commonest cause of preventable mental retardation. The incidence of CH is estimated as one per 3 000-4 000 newborns (2,3). The most frequent aetiology of primary congenital cases in Spain is thyroid ectopy (40.3%), followed by thyroid agenesis (36.7%), dyshormonogenesis (11.8%) and hypoplasia (3.4%) (4). Though mostly sporadic, thyroid dysgenesis (ectopy, agenesis and hypoplasia) is attributed to alteration of the constitution or function of genes involved in thyroid development (4). In 3-4% of cases, the gene affected encodes one of the four transcription factors, the joint presence of which defines thyroid follicular cells and their precursors from the very beginning of thyroid morphogenesis: TITF1/NKX2-1, TITF2/FOXE1, PAX8 or HHEX (4,5,6). Since in the normal individual none of these factors is exclusive to the thyroid, extrathyroidal alterations arise in hereditary cases (7,8,9).

The gene for TITF1/NKX2-1 is located on chromosome 14 in locus q13 in the thyrocytes of the healthy developed  thyroid, this protein regulates the genes for Tg and thyroperoxidase (both involved in T4 synthesis) by binding to their promoter regions. It also plays crucial developmental and functional roles in the brain and lung. In the lung, it controls the expression of surfactant proteins and the morphogenic protein Bmp4, among others (10), while in the brain, it is essential for the formation of the basal ganglia, the migration of GABA neurons to the cortex, and other processes (11,12). Accordingly, most reported cases of haploinsufficient patients with heterozygous mutation have involved mild thyroid dysfunction together with both neonatal respiratory difficulties and choreoathetosis (13,14,15,16,17), although the precise combination of symptoms can depend on the specific mutation. In one report, a heterozygotic 825delC defect gave rise to hypothyroidism and choreoathetosis but no respiratory distress (18). There have been no reports of humans with mutations of both alleles, and it is assumed that like the corresponding mouse models they must die at birth due to pulmonary and/or cerebral defects (5). The fact that in our patient the left thyroid lobe was present but failed to take up contrast suggests that initial morphogenesis was followed by failure of the thyrocytes of that side to differentiate.

The TSH threshold used in screening for CH differs widely among screening centres; the sixteen UK centres, for example, use thresholds ranging from 5 to 12 mIU/L (blood) (19,20), while in the twenty Spanish centres, the threshold is 10 mIU/L (blood). The threshold issue continues to generate considerable debate. Prematurity problems aside, the choice of a threshold depends on the time post partum at which samples are taken, and on screening test technology. Additionally, fears have been expressed that cut-offs as low as 5-10 mIU/L may result in overdiagnosis (21). This criticism seems to overlook several facts: firstly, that a screening test is not a diagnosis; secondly, that a properly handled recall should not throw parents into a state of permanent anxiety, but leave them happy for the extra trouble that is being taken; thirdly, that although moderately depressed, T4 levels may not have the disastrous consequences of classical CH, they are unlikely to be innocuous (22,23); and fourthly, that the lower thresholds currently employed do allow the detection of more cases of classical CH at an acceptable cost in increased recall (20,24). In the present case, moreover, it is possible that the hypothyroid/hypotonic/pulmonary syndrome affecting our patient would not have been identified as soon as it was if hypothyroidism had not initially been detected following a positive screening TSH level of 8.3 mIU/L. Nevertheless, more precise information on the natural history of children with neonatal TSH values in the 5-10 mIU/L range, and on normal ranges of thyroid parameters on a time scale of 24 hours or less, would certainly be welcome (21,24). Lott and coworkers reported central 95% TSH ranges of 0.26-17.1 mIU/L (serum) between 48 and 71 hours post partum, and 0-14.6 mIU/L (serum) between 72 and 95 hours post partum (25).  

As noted above, screening thresholds depend on technology. The TSH threshold was set to 8 mIU/L in Scotland when the DELFIA was introduced (26), and to 5 mIU/L in Wales when the AutoDELFIA method was introduced (19). 

A technical parameter that has previously been taken into account in setting T4 thresholds (see, e.g.), Kempers et al (27), but not TSH thresholds, is within-day variation. Our variable threshold algorithm takes into account both within-day variability and between-day variation in accuracy.

Taking samples at age 5-8 days, as in the UK, is preferable to age 3 days for discrimination between normal infants and those with CH on the basis of TSH measurements, which during this period fall in the former and rise in the latter. If to increase sensitivity still further the default threshold of our algorithm is set to 8 or 9 mIU/L instead of the 10 mIU/L we currently employ, there will be days on which the threshold used nears the 6 mIU/L recommended by Korada and coworkers (20).

In conclusion, patients with CH associated with respiratory pathology and choreoathetosis, ataxia or motor retardation should arouse suspicion of an altered TITF1/NKX2-1 gene. 

In the case discussed here, the use of a TSH screening threshold of just 8.2 mIU/L (whole blood), established by means of our variable threshold algorithm1, allowed 

detection of CH in a patient with a heel-prick TSH level lower than the conventional 10 mIU/L threshold used  worldwide.
